# Curative efficacy of low frequency electrical stimulation in preventing urinary retention after cervical cancer operation

**DOI:** 10.1186/s12957-019-1689-2

**Published:** 2019-08-13

**Authors:** Huan Li, Can-Kun Zhou, Jing Song, Wei-Ying Zhang, Su-Mei Wang, Yi-Ling Gu, Kang Wang, Zhe Ma, Yan Hu, Ai-Min Xiao, Jian-Liu Wang, Rui-Fang Wu

**Affiliations:** 1 Shenzhen Early Diagnosis of Gynecological Major Disease Laboratory, Department of Gynecology, Peking University Shenzhen Hospital, Shenzhen, 518035 Guangdong China; 2 Shenzhen Technical Research and Development Center on Gynecologic Oncology, Shenzhen, 518035 Guangdong China; 3 Department of Obstetrics and Gynecology, Peking University People’s Hospital, No.11 Xizhimen South Street, Xicheng Dist, Beijing, 100044 China; 4 Department of Gynecology, Southern Medical University Affiliated Maternal & Child Health Hospital of Foshan, Foshan, 528000 Guangdong China

**Keywords:** Cervical cancer, Urinary retention, Low-frequency electrical stimulation, Bladder training, Pelvic muscle strength

## Abstract

**Background:**

To evaluate the clinical significance of low-frequency electrical stimulation in preventing urinary retention after radical hysterectomy.

**Methods:**

A total of 91 women with stage IA2–IB2 cervical cancer, who were treated with radical hysterectomy and lymphadenectomy from January 2009 to December 2012, were enrolled into this study and were randomly divided into two groups: trail group (48 cases) and control group (43 cases). Traditional bladder function training and low-frequency electrical stimulation were conducted in the trail group, while patients in the control group were only treated by traditional bladder training. The general condition, rate of urinary retention, and muscle strength grades of pelvic floor muscle in the perioperative period were compared between these two groups.

**Results:**

The incidence of postoperative urinary retention in the electrical stimulation group was 10.41%, significantly lower than that in the control group (44.18%), and the difference was statistically significant (*P* < 0.01). The duration of postoperative fever and use of antibiotics were almost the same between these two groups. Eleven days after surgery, the difference in grades of the pelvic floor muscle between these two groups was not statistically significant. However, 14 days after the operation, grades of the pelvic floor muscle were significantly higher in the trail group than in the control group, and the difference was statistically significant (*P* < 0.01). In addition, although there was no significant difference between the two groups with different parameters (*P* = 0.782), the incidence of urinary retention was lower in the endorphins analgesia program group than in the neuromuscular repair program group (9.09% < 11.54%).

**Conclusion:**

Low-frequency electrical stimulation is more effective than conventional intervention in preventing urinary retention after radical hysterectomy. It also intensifies the recovery of pelvic muscle strength.

## Introduction

Cervical cancer is the most common gynecologic malignant tumor, and extensive panhysterectomy is a radical surgery for the treatment of cervical cancer. The wide range of surgical resections (including the uterus, fallopian tube, the upper part of the vagina, the main ligament, and the paracolpium) and the complete dissection of the pelvic lymph nodes often brings a number of postoperative complications such as bladder dysfunction (anesthesia, urinary incontinence, and urinary retention), lymph cyst, lymphedema, ureterovaginal fistula, intestinal obstruction, wound infection, and urinary tract infections, in which bladder dysfunction is the most common [1]. The incidence of bladder dysfunctions after radical hysterectomy for cervical cancer is 8.0–80% [2, 3]. Postoperative urinary retention can lead to the excessive expansion of the bladder or even permanent detrusor damage, and preventing urinary retention through an indwelling catheter may bring pain and inconvenience to the patient, increasing the incidence of urinary tract infection [4, 5]. In recent years, some scholars have begun to apply electrical stimulation for postoperative rehabilitation of malignant tumors, effectively improving the quality of life of patients. Mariotti et al. randomly divided 60 patients with prostate cancer into two groups according to 1:1. The treatment group received pelvic floor electrical stimulation on the seventh day after the operation, and the control group received routine nursing treatment. Postoperative follow-up showed that the urination function of the treatment group was significantly better than that of the control group [6]. In terms of gynecologic malignancies, Eun et al. randomized 34 patients with gynecologic malignancies to two groups; the electrical stimulation group received 4 weeks of electrical stimulation, and the control group received conventional treatment. The quality of life questionnaire showed that the quality of life and pelvic floor function of the patients in the electrical stimulation group were significantly better than those in the control group [7]. At present, there are few studies on the rehabilitation treatment of urinary retention electrical stimulation after cervical cancer surgery. Based on the previous studies, we will fill in the gaps in related research fields and provide effective treatment methods for improving the postoperative quality of life of patients with cervical cancer.

## Methods

### General information

A total of 91 patients with stage IA2–IB2 cervical cancer, who underwent extensive panhysterectomy and pelvic lymph node dissection in our department from January 2009 to December 2012, were enrolled into the study and were randomly divided into two groups through the closed-envelop method by a person who did not participate in the study: electrical stimulation treatment group (trial group) and control group. There were 48 patients in the trial group and 43 patients in the control group. The researchers who collected the data were unaware of the assignment of participants. The inclusion criteria are as follows: (1) age 18–60 years old, (2) type III hysterectomy for cervical cancer, (3) postoperative pathology indicated that the cancer was completely removed and without distant metastasis, and (4) the patient agreed to conduct the study and sign an informed consent form. The exclusion criteria are as follows: (1) patients with intraoperative urinary tract injury (except ureteral injury), (2) preoperative diagnosis of moderate or above stress urinary incontinence (diaper pad test a 10 g), (3) preoperative urinary retention, and (4) those with uncontrolled epilepsy, central nervous system diseases, mental disorders, or with pacemakers.

### Therapeutic methods

Patients in the two groups underwent bladder function training [8]. Specific operations were as follows. From the 11th day after surgery, between 8 a.m. and 10 p.m., the catheter was opened every 2 h for 15 min each time, while the catheter was fully opened from 10 p.m. to 8 a.m. the next day, until the catheter was pulled out. From the 11th day since the operation, patients in the trial group were given electrical stimulation treatment and bladder function training for 3 days using the Phenix VBS neuromuscular stimulation therapy system (France) [9]; the system is based on the Glazer Protocol, which was originally proposed in 1997 to provide not only a fixed sequence of muscle action and its measurements, but also a database of sEMG readings for normal subjects and pelvic floor dysfunction patients. In the electrical stimulation treatment group, the patient was placed in a supine position, one piece of the electrode was placed in the bladder area under the symphysis pubis, and the other piece was placed on the bone. Two sets of different parameters were chosen according to different treatment principles: electrical stimulation frequency was 35 Hz and pulse width was 200 μs (neuromuscular repair program, group A) or frequency was 1 Hz and pulse width was 270 μs (endorphins analgesia program, group B), for 15 to 30 min each time, twice a day for 3 days. Patients in the control group only received bladder function training for 3 days.

The catheters were removed from all patients in the two groups at 14 days after the surgery. On the same day, residual urine volumes in bladders were measured by B ultrasound to observe whether urinary retention occurred. At the same time, pelvic floor muscle strength was also determined.

Bladder residual urine volume refers to the amount of urine saved in the bladder after urination. This is one of the important indicators for determining bladder urination function:

Urinary retention is when the patient cannot urinate smoothly, bladder residual urine volume was ≥ 100 ml as determined by B ultrasound, or cannot self-discharge and need to reset the catheter.

### Determination of pelvic floor muscle strength by manual muscle test (MMT) [10]

Pelvic floor muscle strength was determined in all patients by the same experienced and qualified doctor of Obstetrics and Gynecology. The doctor placed two fingers in the patient’s posterior vaginal wall 1.5 cm from the fornix, with fingers closely contacting with the vaginal wall, guiding the patient to contract the pelvic floor muscles according to instructions. Then, fingers were placed in the patient’s perineum, and in the same way, patients were guided to contract the pelvic floor muscles according to instructions. This would assess the type I and II muscle fibers of shallow and deep pelvic floor muscles, respectively, in order to understand the strength of the pelvic floor muscles. The conditions of the muscle contraction of patients were graded, and the hierarchical description of the test was based on the GRRUG method, namely, O, I, II, III, IV, and V:

Level 0: no sense of muscle contraction is felt;

Level I: the fibrillation of the muscle is felt;

Level II: incomplete contraction of the muscles if felt, which persist for two seconds or contraction-relaxation movement can finish two times;

Level III: complete contraction of muscles without resistance is felt, persisting for three seconds or contraction-relaxation movement can finish three times;

Level IV: complete contraction of muscles with slight resistance is felt, persisting for four seconds or contraction-relaxation movement can finish four times;

Level V: complete contraction of muscles with strong resistance is felt, persisting for five or more seconds or contraction-relaxation movement can complete five times.

### Statistics analysis

Data obtained is said to be processed using statistical software SPPS 16.0, chi-square test, *t* test, or rank sum test.

## Results

In general information, the differences in age, culture degree, the number of pregnancies and deliveries, weight, and clinical staging of cervical cancer between patients in these two groups were not statistically significant.

As shown in Table 1, after removal of the catheter, bladder residual urine volume was significantly lower in patients in the electrical stimulation group than in the control group (*F* = 1.087, *P* = 0.000), and the difference was statistically significant.

**Table 1:** Residual urine volume of two groups after removal of catheter

	Residual urine volume (ml)
Bladder training + electrical stimulation group, mean ± SD	56.85 ± 29.44
Bladder training group, mean ± SD	95.79 ± 24.07
F value	1.087
*P* value	0.000

The electrical stimulation group has significantly less residual urine than control group (*F* = 1.087, *P* = 0.000)

As shown in Table 2, the difference in postoperative fever days of patients and antibiotic use days between the two groups was not significant, and *P* values were 0.818 and 0.754, respectively. At the beginning and end of the postoperative treatment intervention (11th and 14th day, respectively), the routine urine test was conducted to observe whether patients developed urinary tract infection and was expressed as the leukocyte count. There was no significant difference in urinary tract infection between the two groups, and *P* values were 0.229 and 0.500, respectively.

**Table 2:** Postoperative general condition of two groups

	Days of fever	Days of antibiotics used	U-LEU 11 days after operation	U-LEU 14 days after operation
Bladder training + electrical stimulation group, median (range)	2 (2–3)	3 (3–4)	3 (2–4)	3 (2–4)
Bladder training group, median (range)	2 (2**–**3)	3(3**–**4)	3(2**–**4)	3(2**–**4)
*Z* value	− 0.43	− 0.347	− 0.704	− 0.112
*P* value	0.667	0.728	0.481	0.911

As shown in Table 3, the incidence of urinary retention was significantly higher in the control group than in the electric stimulation treatment group (*P* = 0.000).

**Table 3:** Postoperative urine retention of two groups

Groups	Urine retention (*N*)	Non-urine retention (*N*)	Total	Incidence rate (%)
Bladder training + electrical stimulation group	5	43	48	10.41
Bladder training group	20	23	43	46.51
Total	25	56	91	27.47

As shown in Table 4, the difference was not statistically significant between the two groups with different parameters (*P* = 0.782), but the incidence of urinary retention in group B was lower than in group A (9.09% < 11.54%). This prompts that the treatment effect in the endorphins analgesia program is better compared with that in the neuromuscular repair program.

**Table 4:** Urine retention after treated by different programs

Groups	Urine retention	Non-urine retention	Total	Incidence rate (%)
Group A	3	23	26	11.54
Group B	2	20	22	9.09
Total	5	43	48	10.41

*Group A* neuromuscular repair program, *group B* endorphins analgesia program

As shown in Table 5, the levels of muscle strength in the group of bladder training and electrical stimulation before and after the intervention underwent paired *t* test. It was found that all types of muscle fibers of the pelvic floor were statistically significant (*P* < 0.05), suggesting that low-frequency electrical stimulation can enhance pelvic floor muscle strength.

**Table 5:** Pelvic muscle strength before and after treatments

	D11qI	D11 qII	D11 sI	D11 sII	D14 qI	D14 qII	D14 sI	D14 sII
Bladder training + electrical stimulation group, mean ± SD	1.98 ± 0.86	2.0 ± 0.74	2.06 ± 0.67	2.10 ± 0.66	2.92 ± 0.58	2.75 ± 0.60	2.98 ± 0.56	3.08 ± 0.58
Bladder training group, mean ± SD	2.05 ± 0.65	2.05 ± 0.53	1.93 ± 0.63	1.91 ± 0.65	2.05 ± 0.58	2 ± 0.58	1.88 ± 0.63	1.98 ± 0.60
*χ*^2^ value	6.630	6.629	8.353	2.080	35.489	27.037	46.086	46.086
*P* value	0.085	0.036	0.039	0.353	0.000	0.000	0.000	0.000

*D* how many days after operation, *q* superficial pelvic muscle, *s* deep pelvic muscle, *I* type I muscle fiber, *II* type II muscle fiber; for example, D11 sII represents deep type II muscle fiber 11 days after operation

## Discussion

When patients with cervical cancer undergo extensive panhysterectomy and pelvic lymph node dissection, some of them would develop urinary retention. The reason is damage to the pelvic fascia, ligaments, pelvic support tissue, blood vessels, lymphatic vessels, and nerves during the operation, resulting in bladder dysfunction; thus, urinary retention is one of the earliest and most common causes of great sufferings. Its pathogenetic mechanism is as follows. The inferior hypogastric plexus bladder branch contains sympathetic and parasympathetic nerve fibers, which controls the sphincter and detrusor of the bladder, respectively, perceives bladder volume changes, and mutually regulates this to control urination. Parasympathetic nerve injury can reduce bladder sensitivity to pressure, leading to urinary retention. Thus, damage to the pelvic floor support structure leads to a downward position of the vaginal fornix and urethra and emptiness behind the bladder. The bladder in the filling state falls back due to the loss of support strength, excessively stretching towards the sacrum fossa; thus, the bottom of the bladder and posterior length of the urethra forms an acute angle, resulting in the accumulation of urine in the bladder. Bladder neck obstruction can also easily lead to urinary retention. Some patients develop pelvic inflammation and adhesion, which result in muscle tension and failure to urinate, leading to varying degrees of urination disorders [11–14].

In the 1970s, Tanagho et al. first applied the electrical nerve stimulation technique in the treatment of patients with bladder dysfunction through direct electrical stimulation on neural pathways influencing the bladder detrusor, promoting the recovery of sacral nerve function, which adjusts bladder function [15]. In 1997, sacral nerve electrical stimulation treatment was certificated by the FDA for the treatment of unstable bladder and non-obstructive urinary retention [16]. Peters et al*.* reported that in the treatment of primary urinary retention using sacral nerve stimulation, improvement of symptoms was up to 73% [17]. Kajbafzadeh et al. used transcutaneous functional electrical stimulation (FES) to treat children with refractory neurogenic urinary incontinence secondary to myelomeningocele (MMC) and found that FES was a safe, non-invasive, and effective method compared with the control group [18]. The possible mechanism may be as follows. The second, third, and fourth sacral nerves control bladder function, in which these nerves grow from the root of the third sacral nerve and are mainly distributed in the detrusor of the bladder and pelvic floor levator ani muscle. The root of the third sacral nerve contains the sensory plexus from the pelvic floor and motor plexus from the detrusor (parasympathetic), as well as motor plexuses from the sphincter and other muscles. Effective electrical stimulation on the third sacral nerve can provide a curative effect through the regulation of the urination reflex mediated by the afferent nerve, promoting the recovery of bladder sensation and establishing a normal urination reflex at the same time. In recent years, many studies have reported that after surgery, pelvic floor nerve electrical stimulation could promote the recovery of bladder function, which brought a good curative effect [19, 20].

In the past, in order to avoid the occurrence of urinary retention, the common method was as follows: 3 days of bladder function training was performed before the removal of the catheter on the 14th day after surgery, but this effect was limited. Through the randomized controlled study, our hospital data revealed that the incidence of urinary retention in the control group was 46.51%. From the above treatment principle, the following treatment method was adopted in our hospital: in addition to bladder function training 3 days before the removal of the catheter, low-frequency electric stimulation treatment is performed. This treatment method significantly reduced the incidence of urinary retention, reaching 10.41%; which indicate that low-frequency electric stimulation also can significantly reduce the incidence of urinary retention after cervical cancer operation. This is of great practical significance to the rehabilitation and improvement of the quality of life after surgery.

For the treatment of cervical cancer patients with urinary retention after extensive panhysterectomy, the principle of low-frequency electrical stimulation is as follows. Through electrodes placed on abdominal skin, pulsed current functions as a stimulating factor that strengthens pelvic floor muscles, making the bladder muscles move and causing contraction of the detrusor muscle and relaxation of the urethral sphincter; thus, urination occurs. Data in our hospital revealed that the pelvic floor muscle strength of patients was greatly strengthened after low-frequency electrical stimulation treatment. In addition, low-frequency electrical stimulation also improved the blood circulation of pelvic tissues (including the bladder and urethra), promoting the healing of surgical trauma. Moreover, pulse current stimulation to the nerve controlling bladder can also accelerate the recovery of nerves controlling the bladder and urethra [21].

There are two mechanisms for the treatment of postoperative nerve injury. One is urinary retention caused by bladder detrusor dysfunction, and the main treatment is to recover the activity of the detrusor. Since the internal sphincter and bladder detrusor are controlled by the sympathetic and parasympathetic nerves and in urine activity, the role of sympathetic nerves is second and the parasympathetic nerve plays a dominant role, while pelvic nerves originate from sacral spinal nerves 2–4, which is rich of parasympathetic fibers, in which excitement can make the detrusor contract and the internal bladder sphincter relax, promoting urination [22, 23]. Therefore, the treatment is as follows: two electrodes are connected to the A1 channel of the neuromuscular stimulation therapy system (Phenix), electrodes are placed in the position of sacral spinal nerve 3, and the bone electrode is attached to the bone; the frequency is set at 35 Hz, pulse width at 200 μs, for 15–30 min each treatment, twice a day, for a duration of 3 days, and then, the catheter is removed. The second treatment is the use of endorphin procedures to relax abdominal and pelvic floor muscles with an analgesic effect [24]. In theory, the latter is suitable for muscle tension caused by pain and infection factors after delivery and general surgery [25], and its treatment program is as follows: electrical stimulation frequency is set at 1 Hz, pulse width is set at 270 μs, for 15–30 min each time, twice a day, for a duration of 3 days, then the catheter is removed. However, data of our hospital suggest that in the preventive treatment for urinary retention after cervical cancer operation, the second method seems to be more effective, and the possible reason may be as follows: even the chances of damage to pelvic floor tissues, blood vessels and nerves are greater in cervical cancer operation than in general gynecological surgery, and postoperative urinary retention caused by muscle tension are still dominant. This may be an important discovery in our clinical work.

The female pelvic floor is composed of multi-layer muscle and fascia, supporting and maintaining the pelvic organs in the normal position, in which the inner pelvic diaphragm is the most tough layer of the bottom of the pelvis, including the levator ani muscle and its fascia. Pelvic floor muscle fibers are divided into two types; most of the type I muscle fibers distributed in the levator ani muscle, and type II fibers are located in the superficial pelvic floor.

The pulse current below 1 000 Hz is called low-frequency electrical stimulation. Biological electrical stimulation is the use of low-frequency electromyography to stimulate neuromuscular stimulation. Through passive muscle training, the nociceptors are awakened to accelerate the regeneration of nerve and the recovery of conduction function, stimulate muscle contraction, improve blood circulation, and increase its contraction strength and elasticity, which can enhance pelvic floor muscle strength.

Therefore, pelvic floor electrical stimulation treatment can effectively improve the pelvic floor electrophysiology to repair the damaged neuromuscular; the patient’s pelvic floor muscle strength was significantly improved. The results of this study further indicate that pelvic electrical stimulation can enhance pelvic floor muscle contractility, which is consistent with other studies reported. All in all, through the pelvic floor electrical stimulation treatment, pelvic muscle contraction can effectively improve the function and promote postoperative pelvic floor muscle strength recovery, thereby enhancing the postoperative quality of life.

There are some shortcomings in this study, including the small number of samples included, and non-multi-center research, which makes it difficult to recruit a sufficient number of representative research objects.

## Conclusions

Low frequency electric stimulation is an effective treatment for pelvic floor dysfunction, which has gained attention in recent years, while through observation, we found that it is also significantly effective for urinary retention, non-expensive, non-invasive, easy to operate, its curative effect is not related to age, weight, and so on, and is easily accepted by patients. Thus, it is worthy of clinical promotion. However, low-frequency electrical stimulation treatment for urinary retention after cervical cancer operation remains as a new field of exploration; further assessments of its effect, as well as continuous research and exploration, are required in the future.

## Data Availability

We declared that materials described in the manuscript, including all relevant raw data, will be freely available to any scientist wishing to use them for non-commercial purposes, without breaching participant confidentiality.

## Abbreviations

MMT: Manual muscle test
